# Comment on: effect of pomegranate flower extract oncis platin-induced nephrotoxicity in rats

**DOI:** 10.12860/jnp.2014.23

**Published:** 2014-10-01

**Authors:** Mahmoud Rafieian-Kopaei, Masih Hosseini, Hedayatollah Shirzad

**Affiliations:** ^1^Medical Plants Research Center, Shahrekord University of Medical Sciences, Shahrekord, Iran

**Keywords:** Nephrotoxicity, Cisplatin, Pomegranate

**Implication for health policy/practice/research/medical education:**

Antioxidant supplements have generally beneficial effects, however, several factors such as the kind of the antioxidant and the dose should be considered to avoid the potential negative effects.

## Dear editor

We read with respect the paper “effect of pomegranate flower extract on cisplatin-induced nephrotoxicity in rats” by Motamedi *et al*. (1), in your esteemed journal *“Journal of Nephropathology”*. It is an interesting paper, however, I would like to add some complementary comments to it. Other than antioxidant activity, this paper was designed to investigate the protective effect of pomegranate flower extract (PFE) on cisplatin (CP) induced-renal toxicity. Thirty two male Wistar rats were divided into five groups. The animals in groups 1 to 3 received PFE (25 mg/kg), PFE (50 mg/kg), and placebo (saline), respectively, for 9 days, and from day 3 on, they also received CP (2.5 mg/kg/day). Groups 4 and 5 were treated with PFE (25 and 50 mg/kg/day) alone. Finally, the animals were sacrificed at day 9 after collecting blood samples. The kidneys were removed, weighted, and underwent histological investigation.

The results of the study showed an increase in the mean serum level of creatinine in group 3 (treated with CP and placebo) (p<0.05), but the value decreased significantly (p<0.05) when low dose of PFE was added to CP (group 1). Kidney weight and weight loss in group 1 were non-significantly lower than those in other CP-treated groups (groups 2 and 3). The data from histological staining also indicated less tissue damage in group 1 (when low dose of PFE was added to CP) compared with group 3. They did not observed protective role of PFE in 50 mg/kg against CP-induced toxicity in this animal model.

This effect of pomegranate flower extract was, in part, attributed to the antioxidant activity of the extract. However, surprisingly, pomegranate flower extract at 50 mg/kg had no effect on inhibition of CP-induced nephrotoxicity, while the best effective dose was 25 mg/kg. The high doses of some other antioxidants in other experiments have not shown protective effect, too, and even exacerbated more tissue damage (2).

Here, I would like to explain the possible mechanisms. The involvement of oxidative stress in pathogenesis of CP-induced nephrotoxicity is indisputable. CP-induced renal toxicity is accompanied by production of reactive oxygen species in the kidney. Free radicals have important effects on membrane lipid peroxidation and tissue injury which leads to cell membrane destruction. Free radical production increases the probability of kidney damage.

Biological effects of free radicals in the body are usually controlled by a lot of antioxidants and via antioxidant enzymes like superoxide dismutase (3). Antioxidants beneficial effect is not limited to prevention of kidney damage; they have been shown to predict or ameliorate a wide range of diseases which are supposed to be induced by oxidative stress. Some of these effects include anti-cancer (4), anti-diabetic (5), anti-atherosclerosis (6), antimicrobial (7), and immunomodulatory (8) activities. Antioxidants are also effective in prevention or reduction of a wide range of drugs side effects which are induced by oxidative stress (9-12). However, they may potentially have deleterious effects, too. The major concern about antioxidants supplementation is deleterious effects on reactive oxygen species (ROS) production (pro-oxidant action), especially when precise modulation of ROS levels are needed to allow normal cell function (11,13).

Pro-oxidants are chemical agents that induce oxidative stress, either by generating reactive oxygen species or by inhibiting antioxidant systems. The oxidative stress induced by this agent can damage cells and tissues. In fact, it has been proposed that antioxidants might exhibit pro-oxidant activity in some specific conditions. Of particular importance are dosage, redox condition and the presence of free transition metals in cellular sites. For example, vitamin C, which is an antioxidant, in the presence of ferric iron may act as a potent mediator of lipid peroxidation. It has been suggested that vitamin C may increase DNA damage in humans and similarly β-carotene sometimes act as a pro-oxidant in the lungs of smokers. This pro-oxidant activity is particularly important when a single antioxidant is used (14).

Antioxidants in laboratory examination may dose dependently increase the antioxidant capacity; however, the biological condition is diverse and complex. Therefore, combinations of antioxidant agents that act at multiple metabolic levels are more likely to have beneficial effect (14). Perhaps this is a reason that in traditional medicine several herbal medicine extracts are mixed and used for the treatment of a special disease.

In conclusion, although antioxidant supplements have generally beneficial effects, however, several factors such as the kind of the antioxidant and the dose should be considered to avoid the potential negative effects.

## Authors’ contributions

Manuscript was written by authors equally.

## Conflict of interests

The authors declared no competing interests.

## Funding/Support

None.
